# Supplementary material for: Nuclear Transport Factor 2 (NTF2) suppresses WM983B metastatic melanoma by modifying cell migration, metastasis, and gene expression
Source: Sci Rep. 2021 Dec 8;11:23586. doi: 10.1038/s41598-021-02803-0 (PMC8654834; doi:10.1038/s41598-021-02803-0)
Supplement: Supplementary file 4 — Supplementary Table S4. [file 41598_2021_2803_MOESM4_ESM.docx]

Table S4. List of overlapping DEGs in NTF2 low versus NTF2 high dox+ and NTF2 low versus VGP primary melanoma.

|  | **Ensembl ID** | **Gene ID** |
| --- | --- | --- |
| 1 | ENSG00000003096 | KLHL13 |
| 2 | ENSG00000005513 | SOX8 |
| 3 | ENSG00000015133 | CCDC88C |
| 4 | ENSG00000023839 | ABCC2 |
| 5 | ENSG00000043355 | ZIC2 |
| 6 | ENSG00000058091 | CDK14 |
| 7 | ENSG00000062038 | CDH3 |
| 8 | ENSG00000065717 | TLE2 |
| 9 | ENSG00000065833 | ME1 |
| 10 | ENSG00000066056 | TIE1 |
| 11 | ENSG00000067445 | TRO |
| 12 | ENSG00000070601 | FRMPD1 |
| 13 | ENSG00000070886 | EPHA8 |
| 14 | ENSG00000073464 | CLCN4 |
| 15 | ENSG00000073756 | PTGS2 |
| 16 | ENSG00000074047 | GLI2 |
| 17 | ENSG00000074527 | NTN4 |
| 18 | ENSG00000075618 | FSCN1 |
| 19 | ENSG00000078081 | LAMP3 |
| 20 | ENSG00000080511 | RDH8 |
| 21 | ENSG00000081148 | IMPG2 |
| 22 | ENSG00000081479 | LRP2 |
| 23 | ENSG00000085276 | MECOM |
| 24 | ENSG00000090104 | RGS1 |
| 25 | ENSG00000091428 | RAPGEF4 |
| 26 | ENSG00000091513 | TF |
| 27 | ENSG00000091592 | NLRP1 |
| 28 | ENSG00000091664 | SLC17A6 |
| 29 | ENSG00000099250 | NRP1 |
| 30 | ENSG00000099864 | PALM |
| 31 | ENSG00000099953 | MMP11 |
| 32 | ENSG00000100077 | GRK3 |
| 33 | ENSG00000100167 | SEPT3 |
| 34 | ENSG00000100302 | RASD2 |
| 35 | ENSG00000100351 | GRAP2 |
| 36 | ENSG00000100867 | DHRS2 |
| 37 | ENSG00000101846 | STS |
| 38 | ENSG00000102313 | ITIH6 |
| 39 | ENSG00000102575 | ACP5 |
| 40 | ENSG00000103528 | SYT17 |
| 41 | ENSG00000103647 | CORO2B |
| 42 | ENSG00000104055 | TGM5 |
| 43 | ENSG00000104419 | NDRG1 |
| 44 | ENSG00000104870 | FCGRT |
| 45 | ENSG00000105679 | GAPDHS |
| 46 | ENSG00000105963 | ADAP1 |
| 47 | ENSG00000106066 | CPVL |
| 48 | ENSG00000106069 | CHN2 |
| 49 | ENSG00000106278 | PTPRZ1 |
| 50 | ENSG00000106333 | PCOLCE |
| 51 | ENSG00000107165 | TYRP1 |
| 52 | ENSG00000107242 | PIP5K1B |
| 53 | ENSG00000109089 | CDR2L |
| 54 | ENSG00000109846 | CRYAB |
| 55 | ENSG00000110318 | CEP126 |
| 56 | ENSG00000110328 | GALNT18 |
| 57 | ENSG00000111335 | OAS2 |
| 58 | ENSG00000111371 | SLC38A1 |
| 59 | ENSG00000111490 | TBC1D30 |
| 60 | ENSG00000111879 | FAM184A |
| 61 | ENSG00000112319 | EYA4 |
| 62 | ENSG00000113739 | STC2 |
| 63 | ENSG00000114251 | WNT5A |
| 64 | ENSG00000114270 | COL7A1 |
| 65 | ENSG00000114378 | HYAL1 |
| 66 | ENSG00000115008 | IL1A |
| 67 | ENSG00000115226 | FNDC4 |
| 68 | ENSG00000115363 | EVA1A |
| 69 | ENSG00000115457 | IGFBP2 |
| 70 | ENSG00000115468 | EFHD1 |
| 71 | ENSG00000115604 | IL18R1 |
| 72 | ENSG00000116141 | MARK1 |
| 73 | ENSG00000116833 | NR5A2 |
| 74 | ENSG00000116852 | KIF21B |
| 75 | ENSG00000117115 | PADI2 |
| 76 | ENSG00000117122 | MFAP2 |
| 77 | ENSG00000118407 | FILIP1 |
| 78 | ENSG00000118432 | CNR1 |
| 79 | ENSG00000118495 | PLAGL1 |
| 80 | ENSG00000118640 | VAMP8 |
| 81 | ENSG00000119919 | NKX2-3 |
| 82 | ENSG00000120049 | KCNIP2 |
| 83 | ENSG00000120915 | EPHX2 |
| 84 | ENSG00000121236 | TRIM6 |
| 85 | ENSG00000121361 | KCNJ8 |
| 86 | ENSG00000122367 | LDB3 |
| 87 | ENSG00000122641 | INHBA |
| 88 | ENSG00000122733 | PHF24 |
| 89 | ENSG00000122877 | EGR2 |
| 90 | ENSG00000122986 | HVCN1 |
| 91 | ENSG00000124126 | PREX1 |
| 92 | ENSG00000125531 | FNDC11 |
| 93 | ENSG00000126895 | AVPR2 |
| 94 | ENSG00000128242 | GAL3ST1 |
| 95 | ENSG00000128266 | GNAZ |
| 96 | ENSG00000128408 | RIBC2 |
| 97 | ENSG00000128567 | PODXL |
| 98 | ENSG00000128645 | HOXD1 |
| 99 | ENSG00000130038 | CRACR2A |
| 100 | ENSG00000130600 | H19 |
| 101 | ENSG00000130829 | DUSP9 |
| 102 | ENSG00000131094 | C1QL1 |
| 103 | ENSG00000131409 | LRRC4B |
| 104 | ENSG00000131435 | PDLIM4 |
| 105 | ENSG00000131459 | GFPT2 |
| 106 | ENSG00000131797 | CLUHP3 |
| 107 | ENSG00000132026 | RTBDN |
| 108 | ENSG00000132329 | RAMP1 |
| 109 | ENSG00000132623 | ANKEF1 |
| 110 | ENSG00000132639 | SNAP25 |
| 111 | ENSG00000132932 | ATP8A2 |
| 112 | ENSG00000133124 | IRS4 |
| 113 | ENSG00000133134 | BEX2 |
| 114 | ENSG00000133135 | RNF128 |
| 115 | ENSG00000134215 | VAV3 |
| 116 | ENSG00000134326 | CMPK2 |
| 117 | ENSG00000134516 | DOCK2 |
| 118 | ENSG00000134827 | TCN1 |
| 119 | ENSG00000134874 | DZIP1 |
| 120 | ENSG00000135324 | MRAP2 |
| 121 | ENSG00000135525 | MAP7 |
| 122 | ENSG00000135549 | PKIB |
| 123 | ENSG00000135631 | RAB11FIP5 |
| 124 | ENSG00000136425 | CIB2 |
| 125 | ENSG00000136542 | GALNT5 |
| 126 | ENSG00000136574 | GATA4 |
| 127 | ENSG00000137441 | FGFBP2 |
| 128 | ENSG00000137558 | PI15 |
| 129 | ENSG00000137642 | SORL1 |
| 130 | ENSG00000137868 | STRA6 |
| 131 | ENSG00000137869 | CYP19A1 |
| 132 | ENSG00000137959 | IFI44L |
| 133 | ENSG00000137962 | ARHGAP29 |
| 134 | ENSG00000138075 | ABCG5 |
| 135 | ENSG00000138449 | SLC40A1 |
| 136 | ENSG00000138670 | RASGEF1B |
| 137 | ENSG00000139800 | ZIC5 |
| 138 | ENSG00000140092 | FBLN5 |
| 139 | ENSG00000140398 | NEIL1 |
| 140 | ENSG00000141506 | PIK3R5 |
| 141 | ENSG00000143127 | ITGA10 |
| 142 | ENSG00000143382 | ADAMTSL4 |
| 143 | ENSG00000143387 | CTSK |
| 144 | ENSG00000144278 | GALNT13 |
| 145 | ENSG00000144668 | ITGA9 |
| 146 | ENSG00000145358 | DDIT4L |
| 147 | ENSG00000145777 | TSLP |
| 148 | ENSG00000146147 | MLIP |
| 149 | ENSG00000146674 | IGFBP3 |
| 150 | ENSG00000148288 | GBGT1 |
| 151 | ENSG00000148468 | FAM171A1 |
| 152 | ENSG00000148516 | ZEB1 |
| 153 | ENSG00000149294 | NCAM1 |
| 154 | ENSG00000151150 | ANK3 |
| 155 | ENSG00000151276 | MAGI1 |
| 156 | ENSG00000151338 | MIPOL1 |
| 157 | ENSG00000152192 | POU4F1 |
| 158 | ENSG00000152217 | SETBP1 |
| 159 | ENSG00000152402 | GUCY1A2 |
| 160 | ENSG00000152932 | RAB3C |
| 161 | ENSG00000153132 | CLGN |
| 162 | ENSG00000153208 | MERTK |
| 163 | ENSG00000154262 | ABCA6 |
| 164 | ENSG00000154274 | C4orf19 |
| 165 | ENSG00000154358 | OBSCN |
| 166 | ENSG00000154822 | PLCL2 |
| 167 | ENSG00000155886 | SLC24A2 |
| 168 | ENSG00000155974 | GRIP1 |
| 169 | ENSG00000156298 | TSPAN7 |
| 170 | ENSG00000156959 | LHFPL4 |
| 171 | ENSG00000157168 | NRG1 |
| 172 | ENSG00000157214 | STEAP2 |
| 173 | ENSG00000157404 | KIT |
| 174 | ENSG00000158008 | EXTL1 |
| 175 | ENSG00000158270 | COLEC12 |
| 176 | ENSG00000158859 | ADAMTS4 |
| 177 | ENSG00000159263 | SIM2 |
| 178 | ENSG00000160191 | PDE9A |
| 179 | ENSG00000160255 | ITGB2 |
| 180 | ENSG00000160951 | PTGER1 |
| 181 | ENSG00000161249 | DMKN |
| 182 | ENSG00000161544 | CYGB |
| 183 | ENSG00000162444 | RBP7 |
| 184 | ENSG00000162723 | SLAMF9 |
| 185 | ENSG00000162777 | DENND2D |
| 186 | ENSG00000162873 | KLHDC8A |
| 187 | ENSG00000163017 | ACTG2 |
| 188 | ENSG00000163430 | FSTL1 |
| 189 | ENSG00000163568 | AIM2 |
| 190 | ENSG00000164237 | CMBL |
| 191 | ENSG00000164841 | TMEM74 |
| 192 | ENSG00000164929 | BAALC |
| 193 | ENSG00000165124 | SVEP1 |
| 194 | ENSG00000165238 | WNK2 |
| 195 | ENSG00000165246 | NLGN4Y |
| 196 | ENSG00000165259 | HDX |
| 197 | ENSG00000165474 | GJB2 |
| 198 | ENSG00000165548 | TMEM63C |
| 199 | ENSG00000165626 | BEND7 |
| 200 | ENSG00000166145 | SPINT1 |
| 201 | ENSG00000166341 | DCHS1 |
| 202 | ENSG00000166394 | CYB5R2 |
| 203 | ENSG00000166503 | HDGFL3 |
| 204 | ENSG00000166780 | C16orf45 |
| 205 | ENSG00000166924 | NYAP1 |
| 206 | ENSG00000167232 | ZNF91 |
| 207 | ENSG00000167653 | PSCA |
| 208 | ENSG00000167785 | ZNF558 |
| 209 | ENSG00000167815 | PRDX2 |
| 210 | ENSG00000168481 | LGI3 |
| 211 | ENSG00000168621 | GDNF |
| 212 | ENSG00000168843 | FSTL5 |
| 213 | ENSG00000168959 | GRM5 |
| 214 | ENSG00000169083 | AR |
| 215 | ENSG00000169436 | COL22A1 |
| 216 | ENSG00000169515 | CCDC8 |
| 217 | ENSG00000169851 | PCDH7 |
| 218 | ENSG00000169884 | WNT10B |
| 219 | ENSG00000170396 | ZNF804A |
| 220 | ENSG00000170571 | EMB |
| 221 | ENSG00000170667 | RASA4B |
| 222 | ENSG00000170891 | CYTL1 |
| 223 | ENSG00000170962 | PDGFD |
| 224 | ENSG00000171017 | LRRC8E |
| 225 | ENSG00000171033 | PKIA |
| 226 | ENSG00000171094 | ALK |
| 227 | ENSG00000171195 | MUC7 |
| 228 | ENSG00000171208 | NETO2 |
| 229 | ENSG00000171649 | ZIK1 |
| 230 | ENSG00000171757 | LRRC34 |
| 231 | ENSG00000172005 | MAL |
| 232 | ENSG00000172292 | CERS6 |
| 233 | ENSG00000173406 | DAB1 |
| 234 | ENSG00000173705 | SUSD5 |
| 235 | ENSG00000174808 | BTC |
| 236 | ENSG00000174871 | CNIH2 |
| 237 | ENSG00000175356 | SCUBE2 |
| 238 | ENSG00000175899 | A2M |
| 239 | ENSG00000176049 | JAKMIP2 |
| 240 | ENSG00000176907 | TCIM |
| 241 | ENSG00000177464 | GPR4 |
| 242 | ENSG00000177556 | ATOX1 |
| 243 | ENSG00000177606 | JUN |
| 244 | ENSG00000177679 | SRRM3 |
| 245 | ENSG00000178726 | THBD |
| 246 | ENSG00000178814 | OPLAH |
| 247 | ENSG00000179855 | GIPC3 |
| 248 | ENSG00000180071 | ANKRD18A |
| 249 | ENSG00000180190 | TDRP |
| 250 | ENSG00000181577 | C6orf223 |
| 251 | ENSG00000181800 | CELF2-AS1 |
| 252 | ENSG00000182256 | GABRG3 |
| 253 | ENSG00000182326 | C1S |
| 254 | ENSG00000182580 | EPHB3 |
| 255 | ENSG00000182636 | NDN |
| 256 | ENSG00000183087 | GAS6 |
| 257 | ENSG00000183091 | NEB |
| 258 | ENSG00000183098 | GPC6 |
| 259 | ENSG00000183615 | FAM167B |
| 260 | ENSG00000183943 | PRKX |
| 261 | ENSG00000184489 | PTP4A3 |
| 262 | ENSG00000184613 | NELL2 |
| 263 | ENSG00000184702 | SEPT5 |
| 264 | ENSG00000185100 | ADSSL1 |
| 265 | ENSG00000185532 | PRKG1 |
| 266 | ENSG00000185585 | OLFML2A |
| 267 | ENSG00000185668 | POU3F1 |
| 268 | ENSG00000185669 | SNAI3 |
| 269 | ENSG00000185904 | LINC00839 |
| 270 | ENSG00000186297 | GABRA5 |
| 271 | ENSG00000186716 | BCR |
| 272 | ENSG00000187243 | MAGED4B |
| 273 | ENSG00000187608 | ISG15 |
| 274 | ENSG00000187764 | SEMA4D |
| 275 | ENSG00000187773 | FAM69C |
| 276 | ENSG00000187815 | ZFP69 |
| 277 | ENSG00000187955 | COL14A1 |
| 278 | ENSG00000188004 | SNHG28 |
| 279 | ENSG00000188015 | S100A3 |
| 280 | ENSG00000188153 | COL4A5 |
| 281 | ENSG00000188158 | NHS |
| 282 | ENSG00000188171 | ZNF626 |
| 283 | ENSG00000188783 | PRELP |
| 284 | ENSG00000196081 | ZNF724 |
| 285 | ENSG00000196220 | SRGAP3 |
| 286 | ENSG00000196376 | SLC35F1 |
| 287 | ENSG00000196466 | ZNF799 |
| 288 | ENSG00000196632 | WNK3 |
| 289 | ENSG00000197077 | KIAA1671 |
| 290 | ENSG00000197472 | ZNF695 |
| 291 | ENSG00000197632 | SERPINB2 |
| 292 | ENSG00000198121 | LPAR1 |
| 293 | ENSG00000198216 | CACNA1E |
| 294 | ENSG00000198478 | SH3BGRL2 |
| 295 | ENSG00000198542 | ITGBL1 |
| 296 | ENSG00000198682 | PAPSS2 |
| 297 | ENSG00000198780 | FAM169A |
| 298 | ENSG00000198929 | NOS1AP |
| 299 | ENSG00000204116 | CHIC1 |
| 300 | ENSG00000204442 | FAM155A |
| 301 | ENSG00000204860 | FAM201A |
| 302 | ENSG00000204876 | AC021218.1 |
| 303 | ENSG00000205755 | CRLF2 |
| 304 | ENSG00000205777 | GAGE1 |
| 305 | ENSG00000205927 | OLIG2 |
| 306 | ENSG00000205944 | DAZ2 |
| 307 | ENSG00000206190 | ATP10A |
| 308 | ENSG00000206432 | TMEM200C |
| 309 | ENSG00000213401 | MAGEA12 |
| 310 | ENSG00000213626 | LBH |
| 311 | ENSG00000213658 | LAT |
| 312 | ENSG00000214140 | PRCD |
| 313 | ENSG00000214435 | AS3MT |
| 314 | ENSG00000215269 | GAGE12G |
| 315 | ENSG00000221867 | MAGEA3 |
| 316 | ENSG00000221874 | ZNF816-ZNF321P |
| 317 | ENSG00000221887 | HMSD |
| 318 | ENSG00000221994 | ZNF630 |
| 319 | ENSG00000223573 | TINCR |
| 320 | ENSG00000224186 | C5orf66 |
| 321 | ENSG00000225556 | C2CD4D |
| 322 | ENSG00000225649 | AC064875.1 |
| 323 | ENSG00000226232 | NPIPB14P |
| 324 | ENSG00000226835 | AC097059.1 |
| 325 | ENSG00000228623 | ZNF883 |
| 326 | ENSG00000231419 | LINC00689 |
| 327 | ENSG00000232504 | ST3GAL5-AS1 |
| 328 | ENSG00000234444 | ZNF736 |
| 329 | ENSG00000235872 | AC078777.1 |
| 330 | ENSG00000236362 | GAGE12F |
| 331 | ENSG00000236609 | ZNF853 |
| 332 | ENSG00000236700 | LINC01010 |
| 333 | ENSG00000237289 | CKMT1B |
| 334 | ENSG00000239653 | PSMD6-AS2 |
| 335 | ENSG00000242715 | CCDC169 |
| 336 | ENSG00000243137 | PSG4 |
| 337 | ENSG00000248360 | LINC00504 |
| 338 | ENSG00000249341 | AC124017.1 |
| 339 | ENSG00000249378 | LINC01060 |
| 340 | ENSG00000251191 | LINC00589 |
| 341 | ENSG00000251493 | FOXD1 |
| 342 | ENSG00000253379 | RP11-1102P16.1 |
| 343 | ENSG00000253706 | AC011632.1 |
| 344 | ENSG00000255526 | NEDD8-MDP1 |
| 345 | ENSG00000255690 | TRIL |
| 346 | ENSG00000256124 | LINC01152 |
| 347 | ENSG00000257743 | MGAM2 |
| 348 | ENSG00000258461 | AC012651.1 |
| 349 | ENSG00000258610 | AF111169.1 |
| 350 | ENSG00000259417 | CTXND1 |
| 351 | ENSG00000260577 | AC126773.2 |
| 352 | ENSG00000260691 | ANKRD20A1 |
| 353 | ENSG00000261195 | AC027130.1 |
| 354 | ENSG00000262223 | AC110285.1 |
| 355 | ENSG00000267059 | AC005943.1 |
| 356 | ENSG00000267279 | AC090409.1 |
| 357 | ENSG00000267508 | ZNF285 |
| 358 | ENSG00000267696 | ERVK-28 |
| 359 | ENSG00000273608 | SRCIN1 |
| 360 | ENSG00000274274 | GAGE13 |
| 361 | ENSG00000275221 | HIST1H2AK |
| 362 | ENSG00000276649 | AL117335.1 |
| 363 | ENSG00000277147 | LINC00869 |
| 364 | ENSG00000277268 | LHX1-DT |
| 365 | ENSG00000277586 | NEFL |
