# Supplementary material for: Nuclear Transport Factor 2 (NTF2) suppresses WM983B metastatic melanoma by modifying cell migration, metastasis, and gene expression
Source: Sci Rep. 2021 Dec 8;11:23586. doi: 10.1038/s41598-021-02803-0 (PMC8654834; doi:10.1038/s41598-021-02803-0)
Supplement: Supplementary file 7 — Supplementary Figures. [file 41598_2021_2803_MOESM7_ESM.docx]

# SUPPLEMENTAL INFORMATION

# Nuclear Transport Factor 2 (NTF2) suppresses WM983B metastatic melanoma by modifying cell migration, metastasis, and gene expression

Lidija D. Vuković, Pan Chen, Sampada Mishra, Karen H. White, Jason P. Gigley, Daniel L. Levy

**SUPPLEMENTAL FIGURES**

**
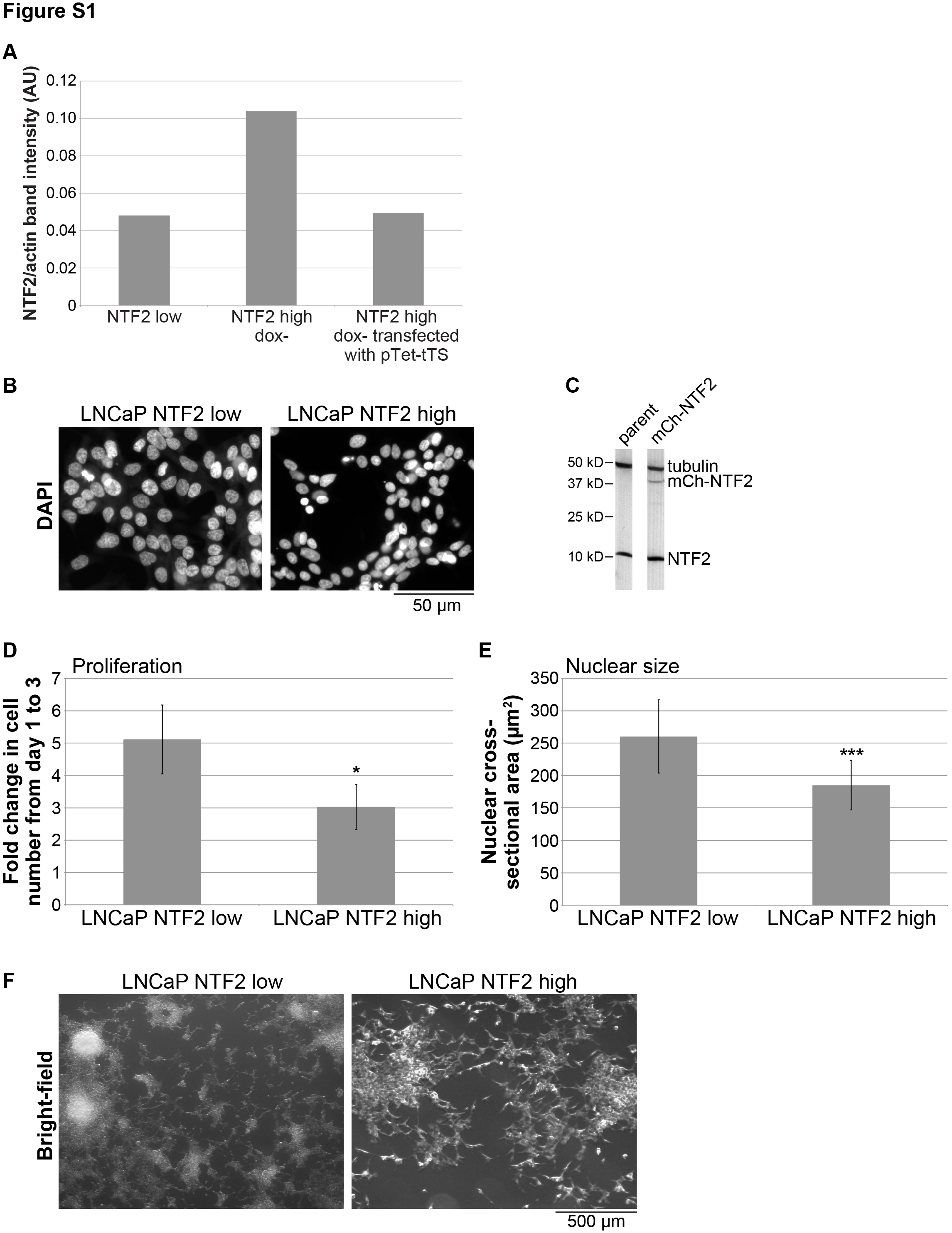
**

**Figure S1: Increasing NTF2 expression in metastatic prostate cancer cells decreases cell proliferation and nuclear size. (A)** Metastatic melanoma cells (WM983B) were stably transfected with a doxycycline-inducible NTF2 construct (pTetOne NTF2). Cell lysates were analyzed by NTF2 and actin immunoblots, as shown in Fig. 1A. For simplicity, “NTF2 low” refers to the parent cell line WM983B and “NTF2 high dox-” refers to the transfected cell line grown without doxycycline. The NTF2 high dox- cell line was transiently transfected with plasmid pTet-tTS to suppress leaky expression of NTF2 from the integrated pTetOne NTF2 construct. NTF2 band intensities were normalized to actin band intensities. Data are shown from one representative experiment. **(B-F)** LNCaP metastatic prostate cancer cells were stably transfected with a construct constitutively expressing mCherry-NTF2. For simplicity, the parent cell line is labeled “LNCaP NTF2 low” and the transfected cell line is labeled “LNCaP NTF2 high.” **(B)** Representative images of DAPI-stained nuclei are shown. **(C)** Cell lysates from the two cell lines were immunoblotted for NTF2 and tubulin. White space delineates cropped lanes from the same blot (Fig. S6B for the uncropped blot). Ectopic expression of mCherry-NTF2 is apparent in the LNCaP NTF2 high cell line. One representative set of samples is shown out of five. On average, mCherry-NTF2 was overexpressed by 19% ± 7% (average ± SD) over endogenous NTF2 levels. **(D)** Cell proliferation was quantified as described in Fig. 1B. The fold change in cell number from day 1 to 3 is plotted for each cell line. Apoptosis rates were not significantly different between the two cell lines. **(E)** Cross-sectional nuclear areas were quantified as described in Fig. 5B. More than 150 nuclei were quantified for each cell line. **(F)** Representative bright-field colony morphology images are shown for each cell line. In the parent cell line “LNCaP NTF2 low,” cells tend to pile on top of each other in colonies, while this is less apparent in the “LNCaP NTF2 high” cell line.

Error bars are SD. Two-tailed Student’s t-tests assuming equal variances with * p < 0.05 and *** p < 0.001.


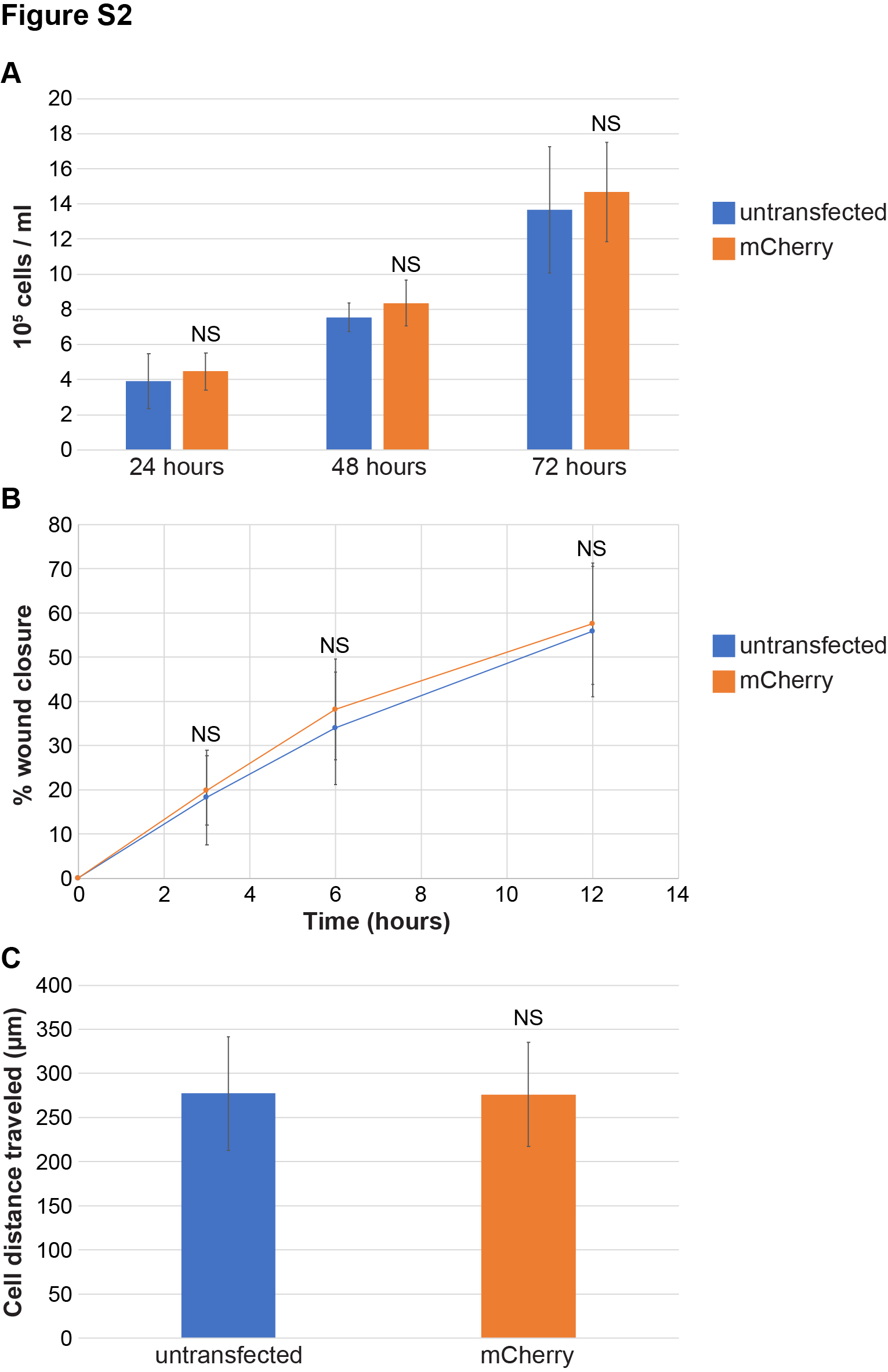


**Figure S2: Generic protein overexpression does not affect cell proliferation or migration in metastatic melanoma cells.** Metastatic melanoma cells (WM983B) were transiently transfected with plasmid pEmCherry-C2 expressing mCherry or no DNA. **(A)** Forty-eight hours post-transfection, 2x10^5^ cells were plated in 6-well plates. At 24, 48, and 72 hours, cells were trypsinized, stained with trypan blue, and counted. Data are from 9 experiments. **(B)** Forty-eight hours post-transfection, wound healing assays were performed by creating large scratches (i.e. P20 pipet tip) through completely confluent cell monolayers and imaging at 0, 3, 6, and 12 hours. The percent wound closure was calculated relative to the scratch area at t=0. Data are from 10-11 experiments. **(C)** The migration trajectories of individual cells in wound healing assays were measured over 12 hours using the MTrackJ plugin in ImageJ. The total distance traveled by 49 untransfected cells and 36 mCherry-expressing cells was quantified. Data are from 4 experiments.

Error bars are SD. NS indicates not significant by two-tailed Student’s t-test assuming equal variances.

**
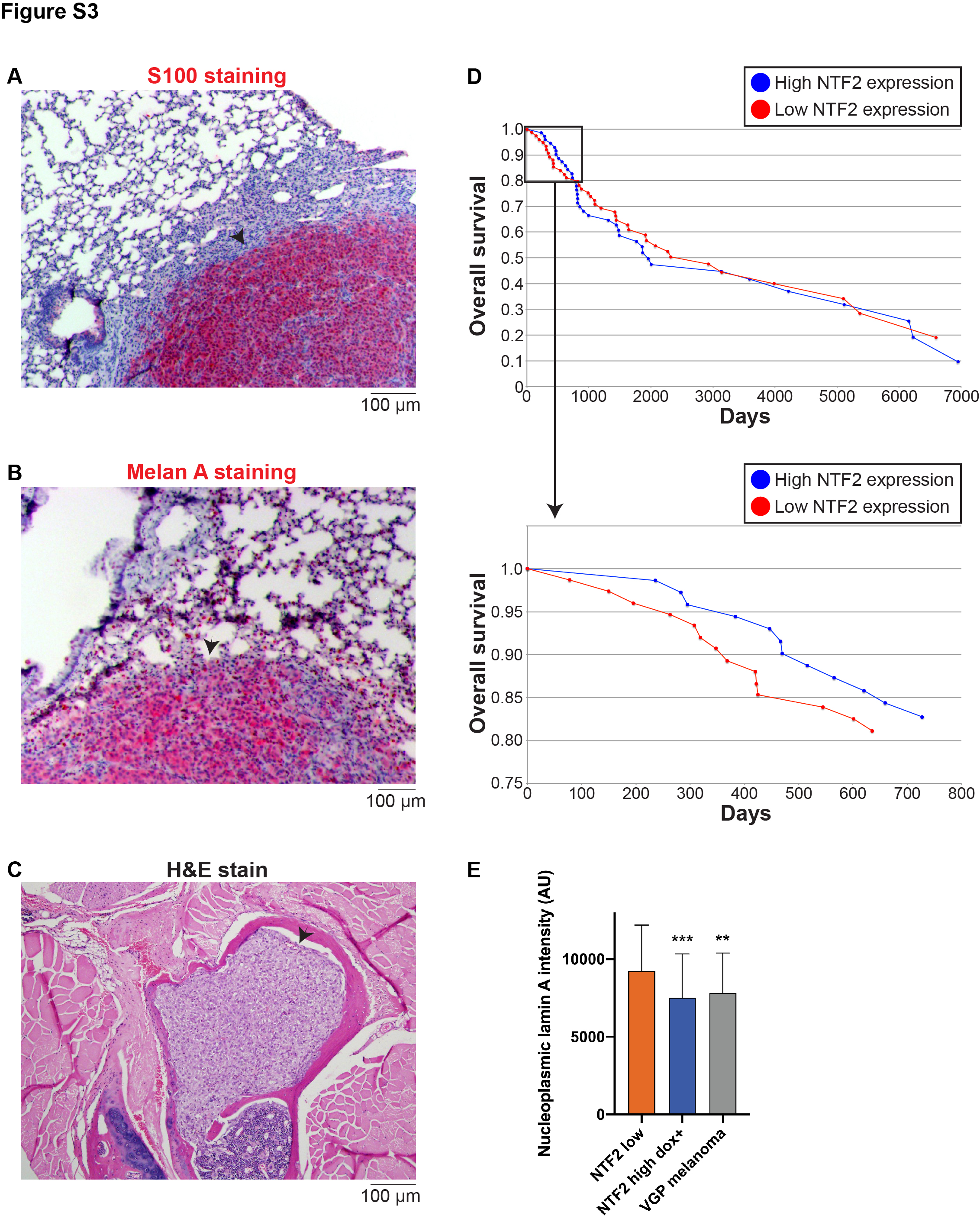
**

**Figure S3: Additional tumor data, TCGA survival data, and nucleoplasmic lamin A data. (A-B)** Rag2^−/−^ γc^−/−^ knockout mice (B6) were injected with NTF2 low melanoma cells. Lung sections from NTF2 low mice were stained for S100 or Melan A. Arrows denote melanoma metastases in the lung that stain positive for S100 or Melan A (red). Also see Figure 3. **(C)** Rag2^−/−^ γc^−/−^ knockout mice (B6) injected with NTF2 low cells infrequently developed spinal cord metastases. An example is shown here, denoted with a black arrow. **(D)** Survival data for patients with metastatic melanoma separated into high and low NTF2 expression groups. These data are from The Cancer Genome Atlas (TCGA) project human skin cutaneous melanoma (SKCM) dataset ([https://www.cancer.gov/tcga](https://www.cancer.gov/about-nci/organization/ccg/research/structural-genomics/tcga)) and were obtained from ^1^. The upper graph shows all data while the lower graph only shows data up to day 728. **(E)** For the experiments described in Fig. 5A, nucleoplasmic lamin A staining intensity was quantified for at least 84 nuclei per condition. Error bars are SD. One-way ANOVA followed by Dunnett’s multiple comparisons test with *** p < 0.0001 and ** p < 0.005.

**
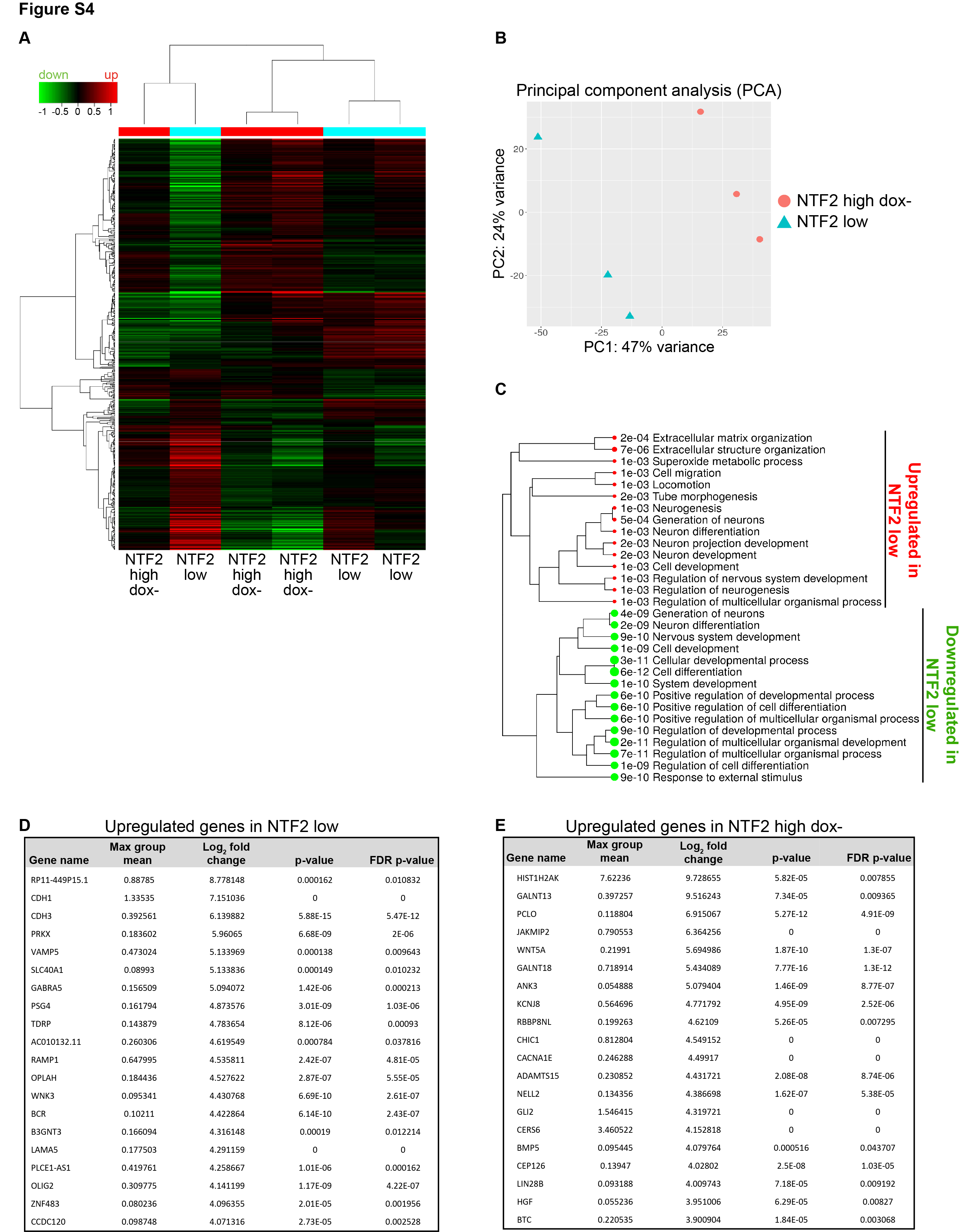
**

**Figure S4: Comparing the transcriptomes of NTF2 low and NTF2 high dox- cells.** RNAseq data were acquired for three independent samples each of NTF2 low and NTF2 high dox- cells. The NTF2 low data are the same described in Fig. 4. **(A)** Expression heat map of the 500 most differentially expressed genes with hierarchical clustering. Red and green represent genes upregulated and downregulated, respectively, relative to a reference transcriptome. The Z score cut-off was 4. **(B)** Two-dimensional principal component analysis (PCA) is shown. **(C)** Enriched pathways are listed for differentially expressed genes. **(D)** Based on log_2_ fold change ranking, the most highly upregulated genes in NTF2 low cells are shown. Cutoff: FDR < 0.05. **(E)** Based on log_2_ fold change ranking, the most highly upregulated genes in NTF2 high dox- cells are shown. Cutoff: FDR < 0.05.


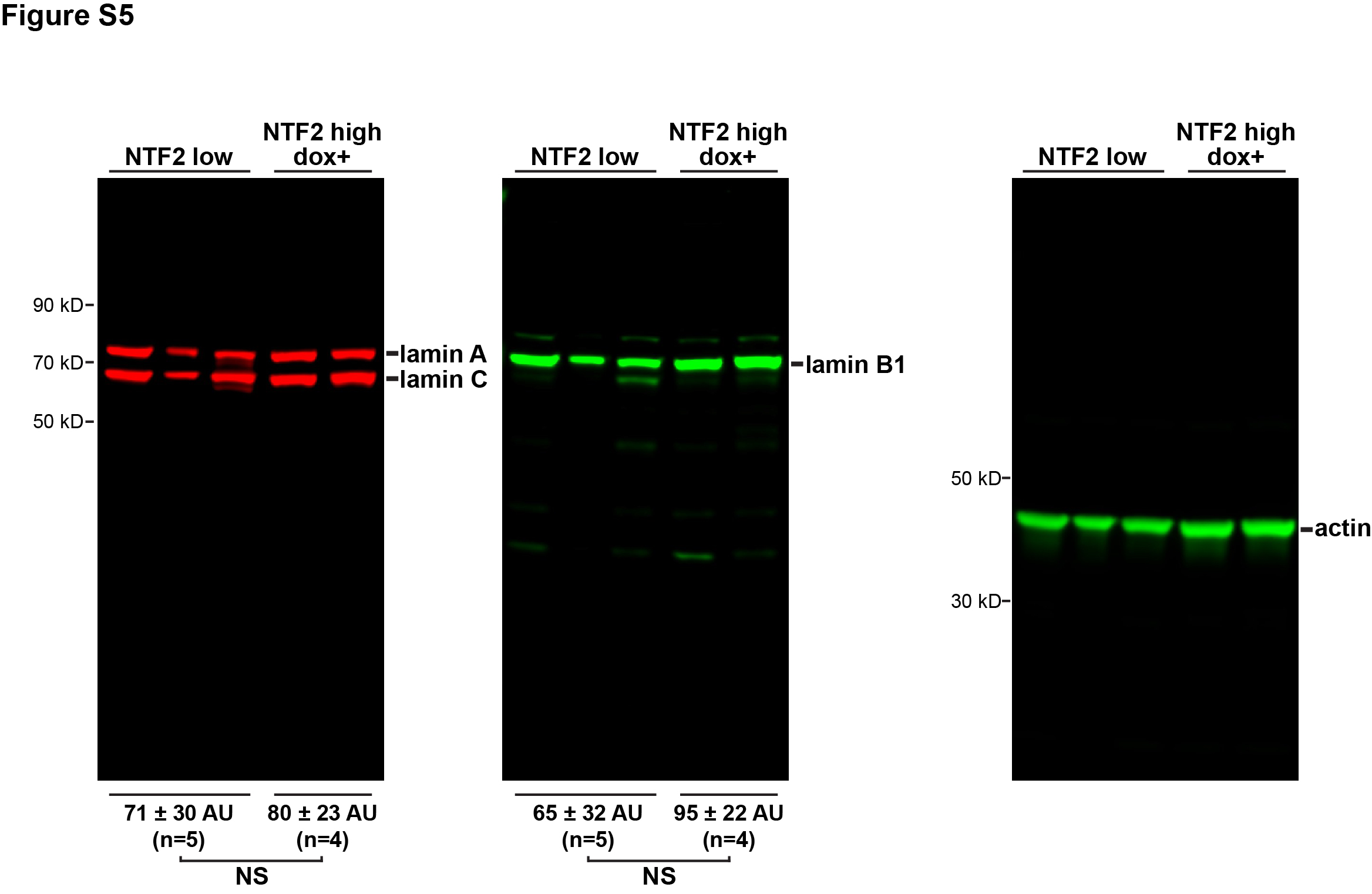


**Figure S5: Lamin A/C and lamin B1 western blots.** NTF2 low and NTF2 high dox+ cell lysates were analyzed by lamin A/C, lamin B1, and actin immunoblots. Lamin A and lamin B1 band intensities were normalized to total protein measured from Ponceau-stained membranes. Average data are shown for 4-5 independent samples per condition. Relative protein amounts are indicated as average ± SD in arbitrary units (AU). Two-tailed Student’s t-tests assuming equal variances with NS not significant.


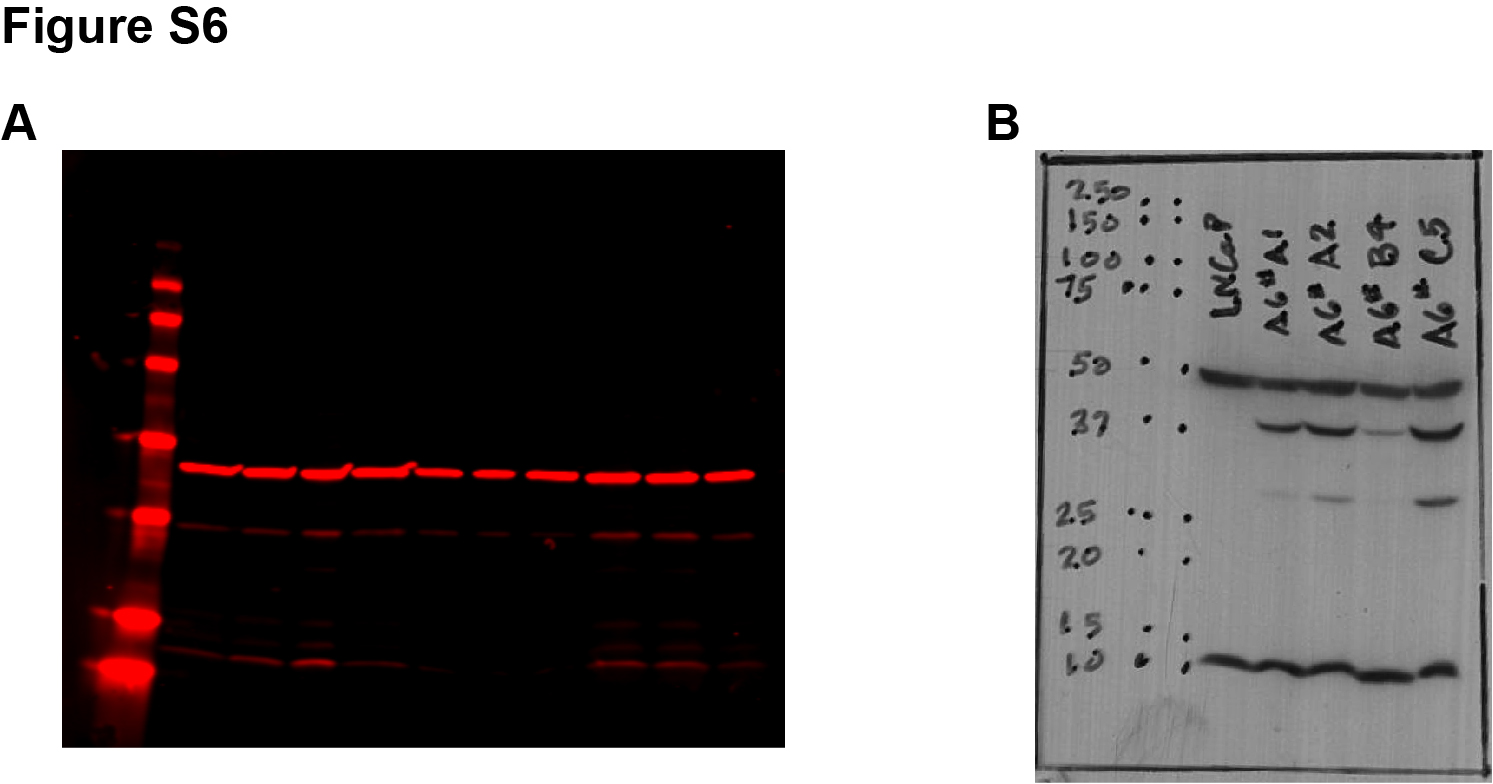


**Figure S6: Uncropped western blots. (A)** Uncropped western blot from Figure 1A. **(B)** Uncropped western blot from Figure S1C.

**SUPPLEMENTAL TABLE LEGENDS**

**Table S1: NTF2 low versus NTF2 high dox+ transcriptomics data.** The RNAseq data are presented for NTF2 low versus NTF2 high dox+. Positive log_2_ fold changes indicate genes upregulated in NTF2 low. Negative log_2_ fold changes indicate genes downregulated in NTF2 low.

**Table S2: NTF2 high dox- versus NTF2 high dox+ transcriptomics data.** The RNAseq data are presented for NTF2 high dox- versus NTF2 high dox+. Positive log_2_ fold changes indicate genes upregulated in NTF2 high dox-. Negative log_2_ fold changes indicate genes downregulated in NTF2 high dox-.

**Table S3: VGP primary melanoma versus NTF2 low transcriptomics data.** The RNAseq data are presented for VGP primary melanoma versus NTF2 low. Positive log_2_ fold changes indicate genes upregulated in VGP primary melanoma. Negative log_2_ fold changes indicate genes downregulated in VGP primary melanoma.

**Table S4: List of overlapping DEGs in NTF2 low versus NTF2 high dox+ and NTF2 low versus VGP primary melanoma.**

**VIDEO LEGENDS**

**Video 1: NTF2 low cells exhibit fast cell migration.** A pipet tip was used to create a scratch through a completely confluent monolayer of NTF2 low cells. Bright-field imaging was performed over twelve hours.

**Video 2: NTF2 high dox+ cells exhibit slow cell migration.** A pipet tip was used to create a scratch through a completely confluent monolayer of NTF2 high dox+ cells. Bright-field imaging was performed over twelve hours.

**REFERENCES**

1 Goswami, C. P. & Nakshatri, H. PROGgeneV2: enhancements on the existing database. *BMC Cancer* **14**, 970 (2014).
